# Rapid-onset acute respiratory distress syndrome after mastectomy in a breast cancer patient: A case report and review of literature

**DOI:** 10.1097/MD.0000000000022795

**Published:** 2020-10-23

**Authors:** Shih-Chao Chien, Shih-Chun Chien, Ting-Yu Hu

**Affiliations:** a Department of Emergency Medicine; b Department of Critical Care Medicine, Mackay Memorial Hospital, Taipei; c Department of Medicine, Mackay Medical College, New Taipei City, Taiwan.

**Keywords:** acute respiratory distress syndrome, cancer, chemotherapy, diffuse alveolar hemorrhage, mastectomy

## Abstract

**Rationale:**

Postoperative acute respiratory distress syndrome (ARDS) often results in severe morbidity and mortality in surgical patients. The etiology of this condition is complex, especially in cancer patients.

**Patient concerns:**

We encountered a 53-year-old woman with left breast cancer, cT1cN2M0, stage IIIA with left axillary lymph node metastasis. She had received chemotherapy with 4 cycles of doxorubicin plus cyclophosphamide, and 4 cycles of trastuzumab plus docetaxel within a span of 6 months. Subsequently, she underwent left simple mastectomy and axillary lymph node dissection, shortly after which she developed respiratory distress with progressive desaturation and hemoptysis.

**Diagnosis:**

ARDS was diagnosed using the Berlin criteria. Her arterial blood gas analysis revealed profound hypoxemia and her chest imaging was suggestive of pulmonary edema. She developed diffuse alveolar hemorrhage (DAH) that was confirmed with bronchoscopy and hemorrhagic samples on bronchoalveolar lavage.

**Interventions:**

She was mechanically ventilated with lung protective measures for management of ARDS. In addition to antibiotic cover with amoxicillin sodium-potassium clavulanate for occult infections during her stay in the intensive care unit, we administered epinephrine inhalations, intravenous treatment with tranexamic acid, and methylprednisolone for DAH.

**Outcomes:**

Her clinical course improved; she was extubated successfully on day 7 and discharged home on day 11.

**Lessons subsections:**

Chemotherapeutic agents may cause pulmonary toxicity through a direct cytotoxic effect or immune-mediated reactions and result in an increased risk of development of ARDS. Furthermore, surgery may trigger a systemic inflammatory response syndrome that can also induce ARDS. In our patient, the development of ARDS was attributed to the combined effects of surgery and chemotherapeutic agents (trastuzumab or docetaxel). When patients undergo major surgery after receiving chemotherapeutic agents, careful consideration is necessary to prevent the development of ARDS.

## 1 Introduction

Acute respiratory distress syndrome (ARDS) is a rare but serious condition that occurs as a consequence of diffuse alveolar injury. Alveolar injury causes the release of inflammatory markers such as interleukin 6 (IL-6) and interleukin 8 (IL-8), and this causes damage to the capillary and alveolar endothelium. The resultant loss of oncotic gradient and surfactant leads to severe adverse effects.^[1]^ ARDS is a clinical diagnosis that is made using the Berlin criteria.^[2]^ ARDS has been managed with multiple treatment strategies including lung protective measures. Despite this, the mortality is still high. Maca et al presented a systemic review of ARDS demonstrating that since 2010, the in-hospital and intensive care unit (ICU) mortality rates were 45% and 38%, respectively.^[3]^

As a result of its poor prognosis, devising strategies to prevent patients from developing ARDS is important. Sepsis, pneumonia, pancreatitis, toxins, and transfusions have been implicated in the etiology of ARDS.^[4]^ In addition, many surgical conditions, major operations, and trauma have been frequently reported as causative factors. Herein, we present a case of postoperative ARDS that developed within a few hours. The combined effects of chemotherapy and stressors of surgery may have contributed to the rapid clinical deterioration of our patient.

## 2 Case presentation

**Figure 1:**
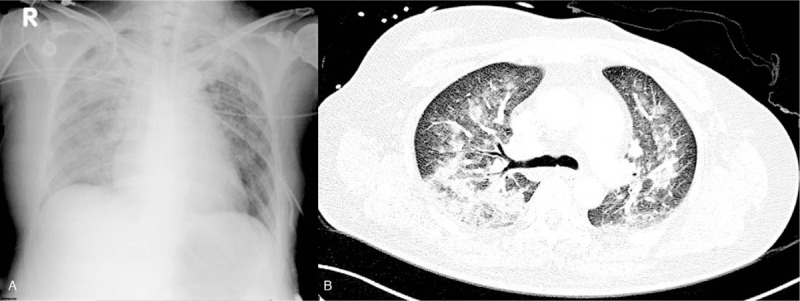
(A) Postoperative plain chest roentgenograms reveal bilateral lung consolidation. (B) Chest computed tomography image (lung window) of the patient with severe diffuse alveolar hemorrhage shows symmetrical bilateral peribronchial alveolar pattern with interspersed ground-glass opacities indicative of pulmonary edema.

We report the case of a 53-year-old Asian woman, a housekeeper, who presented with history of left breast carcinoma, cT1cN2M0, stage IIIA with left axillary lymph node metastasis (estrogen receptor: 50%, progesterone receptor: 20%, and human epidermal growth factor receptor 2: 3+). She had no relevant familial medical history. She had completed 8 cycles of neoadjuvant chemotherapy 1 month prior to presenting to us. The chemotherapy regimen consisted of 4 cycles of doxorubicin plus cyclophosphamide followed by 4 cycles of trastuzumab plus docetaxel with an accumulative dosage (doxorubicin 240 mg/m^2^, cyclophosphamide 2400 mg/m^2^, trastuzumab 1078 mg/m^2^, and docetaxel 317 mg/m^2^). The patient gave history of minimal side effects from chemotherapy, experiencing only mild nausea and vomiting. There was no history of fever, shortness of breath, or other discomfort prior to admission. She was admitted and planned for simple left mastectomy and axillary lymph node dissection. During the operation, the estimated blood loss was minimal at 10 ml, and blood transfusion was not required. The operation time was about 3 hours. In the postoperative recovery room, she vomited out around 10 ml of bloody vomitus while emerging from anesthesia. Her oxyhemoglobin saturation (SpO2), as measured by pulse oximetry, was 83%, and supplemental oxygen via an aerosol mask with FiO2 of 60% was administered. The patient then began to develop progressive dyspnea, and her SpO2 gradually decreased to 70% despite the FiO2 being titrated up to 100% within the next 90 minutes. Chest radiography revealed bilateral pulmonary edema with consolidation (Fig. 1A). The patient was re-intubated and 70 ml of fresh bloody secretions was suctioned through the endotracheal tube. Subsequently, she was transferred to the intensive care unit for further management.

**Figure 2:**
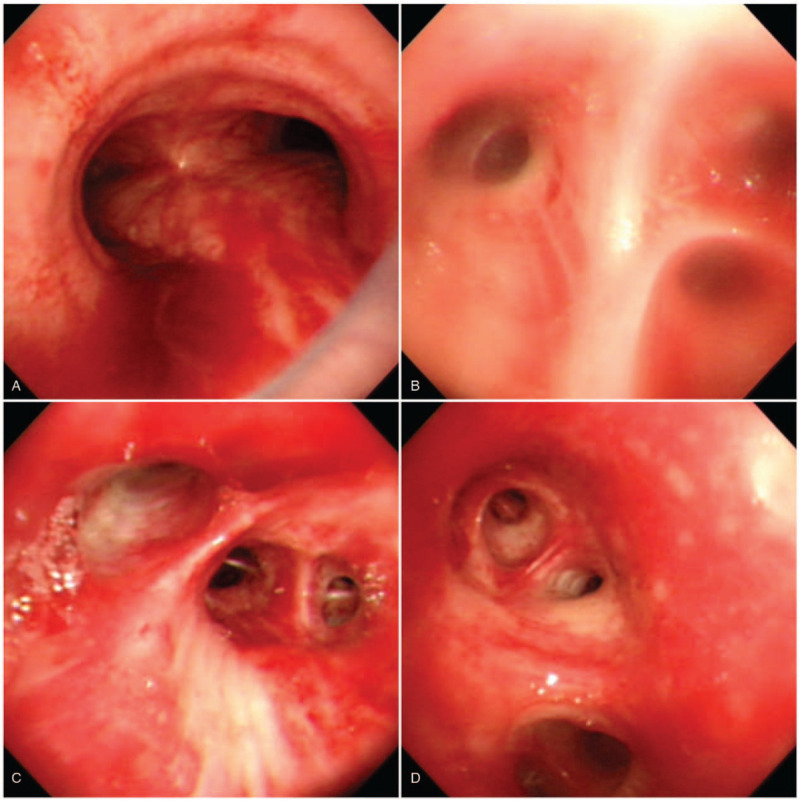
Bronchoscopy findings reveal diffuse bleeding in the bilateral bronchi, particularly in the main carina (A), right upper lobe (right B2 and B3) (B), right middle and right lower lobes (C), left secondary carina, and proximal left bronchi (D).

In the ICU, an extensive examination was performed to determine the cause of the sudden postoperative pulmonary edema. The laboratory reports revealed mildly elevated N-terminal prohormone of natriuretic peptide (NT-proBNP; 346 pg/ml, normal range: 0–125 pg/ml) and elevated D-dimer (1844 ng/ml, normal range: <500 mg/ml), fibrinogen (417.5 mg/dl, normal range: 200.0–400.0 mg/dl), fibrin degeneration product (FDP; 7.5 mg/dl, normal range: <5 mg/dl), and C4 (45 mg/dl, normal range: 16–38 mg/dl) levels. The procalcitonin level was less than 0.05 ng/ml. The autoimmune marker analysis included the estimation of antinuclear antibody (ANA), anti-neutrophil cytoplasmic antibody-perinuclear (P-ANCA), anti-neutrophil cytoplasmic antibody-cytoplasmic (C-ANCA), anti-cyclic citrullinated peptide (anti-CCP), rheumatoid factor (RF), anti-extractable nuclear antigen (anti-ENA), lupus anti-coagulant, C3, and C4, all of which were within the normal ranges. A computed tomography (CT) scan revealed a symmetrical bilateral peribronchial alveolar pattern mixed with ground glass opacities indicative of pulmonary edema. Neither a pulmonary embolism nor an enlarged pulmonary trunk was found (Fig. 1B). Intravenous amoxicillin sodium-potassium clavulanate was administered empirically at 1.2 g every 6 hours in view of the consolidation, as infection could not be excluded at this stage. Tranexamic acid was also administered intravenously, at 1 g every 6 hours, along with 3 ml of 1:1000 epinephrine as inhalation for the hemoptysis. On day 2 in the ICU, fiberoptic bronchoscopy was performed in order to determine the cause of acute pulmonary edema. Diffuse bronchial mucosal hemorrhage with severe erythema and edematous changes were seen throughout the bronchial tree, and a large volume of fresh blood was drained from the right sided bronchi. A bronchoalveolar lavage (BAL) was performed by wedging the bronchoscope into the right upper lobe bronchus where the radiologic opacity was most obvious and the lavage fluid was progressively more hemorrhagic, a characteristic symptom of diffuse alveolar hemorrhage (DAH) (Fig. 2). Therefore, methylprednisolone was prescribed at a dose of 20 mg every 8 hours to treat DAH. The patients hypoxemia and hemoptysis gradually improved. Bacterial cultures, fungal cultures, cytomegalovirus polymerase chain reaction, and tests for the aspergillus antigen were performed using the bronchoalveolar lavage fluid, and all these tests yielded negative results. The patient did not develop fever during her ICU stay. According to the abovementioned examinations and investigations, ARDS was deemed the mostly likely cause of DAH and the acute pulmonary edema was determined to be non-cardiogenic. The prior chemotherapy (trastuzumab and docetaxel) and the surgical procedure were considered to be the causes of the ARDS, due to the possibility of chemotherapy-induced direct cytotoxic effects or immune-mediated reactions of the lung and surgery-associated systemic inflammatory response syndrome.

On day 7 in the ICU, the patient was extubated and transferred to the general ward without further complications. She was finally discharged home, 11 days after admission.

Informed consent was obtained from the patient for the publication of this report and accompanying images.

## 3 Discussion

To our knowledge, this is the first case report on the development of ARDS in a patient who underwent mastectomy for breast cancer. The main point of interest here was the determination of the key factors that led to the development of ARDS. While surgery is known to induce a stress response, there may be many different mechanisms of ARDS development on a case-to-case basis, and these may depend on the type of surgery performed. Boshier et al reviewed the pathophysiology of post-esophagectomy ARDS.^[5]^ The levels of pro-inflammatory cytokines, such as IL-6 and IL-8 were found to be markedly elevated in the lung tissue and pleural fluid. In the lavage, the post-esophagectomy levels of neutrophil elastase, granulocyte-colony stimulating factor, secretory leukocyte protease inhibitor, and nitrogen-free radicals were found to be elevated. In the serum, the levels of elastase and thromboxane A2 were elevated, which caused an increase in the extravascular lung water and a decrease in lung compliance. Additionally, the cumulative effect of different cytokines may induce ARDS. Rong et al speculated that activation of the complement alternative pathway leads to elevated levels of C3a and C5a.^[6]^ The complement effect results in accumulation of activated neutrophils in the pulmonary tissue after cardiopulmonary bypass.^[5,6]^ In our case, there were various risk factors for the development of postoperative ARDS.

The risk factors that are generally reported are as follows:

1. The patients underlying condition including age; current smoking status; presence of medical conditions such as liver cirrhosis, interstitial lung disease, chronic obstructive pulmonary disease (COPD), poor left ventricular ejection fraction (LVEF); New York Heart Association (NYHA) functional class II–IV; previous cardiac surgery; and a history of chemotherapeutic agent use
2. The perioperative events such as large intraoperative crystalloid fluid infusions, massive transfusion, hypertension, hypotension, high levels of oxygen supplementation, and cardiac ischemia.^[5–7]^

The duration and method of surgery are also influencing factors in the development of ARDS. Longer duration of surgery increases the risk of postoperative ARDS and the type of surgery; whether emergent, complex, or open; may result in an elevated rate of ARDS development. Due to the variety of triggers, a lung injury prediction score (LIPS) was developed and has been proven useful in the prediction of the likelihood of developing ARDS.^[4]^ In our case, a simple mastectomy was performed, the surgical time was short, and intraoperatively, the patient remained stable without need for blood transfusion or hemodynamic support and did not display a high oxygen demand. Therefore, the LIPS score of 4 in our patient indicated a relatively low risk ARDS development (a LIPS score ≥7 indicates an increased risk of ARDS in critical care patients following surgery).^[8]^

In our case, following a review of the serial radiological images and laboratory results, the prior use of chemotherapeutic agents was considered as the potential cause of the early postoperative development of ARDS. Doxorubicin and cyclophosphamide were used for the initial 4 cycles, followed by trastuzumab and docetaxel for the next 4 cycles. Review of the available literature led us to conclude that the use of chemotherapeutic agents might have induced the lung injuries. Cyclophosphamide can lead to multiple forms of lung injuries as it causes increased oxidative stress that damages type 1 pneumocytes and induces pulmonary fibrosis in animal models.^[9]^ Further, this agent can induce subacute interstitial lung disease, acute pulmonary edema, organizing pneumonia, and ARDS.^[9,10]^ Docetaxel can induce severe interstitial pneumonitis, ARDS, and fatal respiratory failure.^[10,11]^ Trastuzumab can induce both acute and chronic lung injuries. The acute condition may result from induced bronchospasm and respiratory distress. The chronic condition may lead to bronchiolitis obliterans with organizing pneumonia and interstitial pneumonitis.^[10]^ The onset of ARDS in this case was unexpected and might have had no relation to the duration of the treatment regimen,^[12]^ as ARDS is known to occur immediately after chemotherapy, or several weeks after. Although, the exact mechanism of lung injury in this case is still unknown, we postulate that the subclinical pulmonary toxicities of the chemotherapy might have decreased the threshold for the development of ARDS in this patient.

DAH is frequently found to be associated with autoimmune diseases, such as ANCA-associated vasculitis. Medications, such as hydralazine or allopurinol, may also trigger secondary vasculitis that could induce DAH.^[13]^ Of the chemotherapeutic agents, bevacizumab is presumed to induce DAH through a possible mechanism of inhibition of the vascular endothelial growth factor (VEGF) with resultant fragile capillaries.^[14,15]^ However, bevacizumab had not been used in the treatment for this patient. Ikeda et al discussed the case of a patient who developed DAH after 4 cycles of chemotherapy using a regimen that included bevacizumab (15 mg/kg), cisplatin (80 mg/m^2^), and docetaxel (60 mg/m^2^).^[15]^ Docetaxel was therefore presumed to play a role in inducing pulmonary toxicity in DAH.^[11]^ The exact mechanism of causation requires further research.

Some limitations exist in our case report. First, the patient did not undergo imaging or pulmonary function testing prior to and during chemotherapy. Therefore, clarification of the role of chemotherapy in inducing lung injury prior to surgery could not be obtained. Second, the patient did not undergo autoimmune screening prior to admission, and consequently, an underlying autoimmune disease could not be excluded.

There is progression in the research surrounding treatment of chemotherapeutic induced pulmonary injuries. In recent animal studies, cyclophosphamide-induced acute lung injury may have been alleviated by venlafaxine. Venlafaxine can decrease TNF-alpha, IL-1beta or decrease the activity of inflammatory cells in cyclophosphamide-induced acute lung injury.^[16]^ Curcumin has been shown to downregulate IL-17a and mediate the p53-fibrinolytic system in order to alleviate acute lung injuries due to bleomycin.^[17]^ The efficacy of immunomodulation medication in association with chemotherapeutic agents requires further research for clarification.

## 4 Conclusion

Postoperative ARDS can lead to severe morbidity and mortality. Aside from factors contributing to the surgical stress response, perioperative factors need to be carefully controlled in order to prevent the development of ARDS. Chemotherapeutic agents may result in pulmonary toxicity. Therefore, serial follow-up of pulmonary function may be indicated in high-risk patients, and an imaging survey may be indicated for the diagnosis of subclinical pulmonary complications.

## Acknowledgments

We would like to thank Dr. Yu-Yi Chien for his expert advice and encouragement throughout this study

## Author contributions

Concept and design: Ting-Yu Hu, MD. Drafting of the manuscript: Shih-Chao Chien, MD, and Shih-Chun Chien, MD. Critical revision of the manuscript for important intellectual content: Ting-Yu Hu, MD.
